# Anaesthesia for laparoscopic cholecystectomy in Bartter’s syndrome

**DOI:** 10.4103/0019-5049.68377

**Published:** 2010

**Authors:** Bala S Bhaskar, GV Rao, Sanjeev B Joshi, SK Arun, SK Ajay

**Affiliations:** Department of Anaesthesiology and Critical Care, VIMS, Bellary, India; 1 Pediatric Surgery, VIMS, Bellary, India; 2 SK Panduranga Rao Hospital, Bellary, Karnataka, India

**Keywords:** Anaesthesia, Bartter’s syndrome, hypokalemia, paediatric

## Abstract

Bartter’s syndrome is a rare inherited anamoly with defect in the thick segment of the ascending limb of the loop of Henle, with reduced reabsorption of potassium. Growth is affected with worsening renal function, hypokalaemia, hypochloraemic metabolic alkalosis, hypocalcemia, hypomagnesemia, increased levels of aldosterone, renin and angiotensin without hypertension and lack of responses to vasopressors. Treatment consists of potassium supplementation along with other medications. We present the case report, probably the first, of a child suffering from Bartter’s syndrome with gall stones posted for laparoscopic cholecystectomy. The pre-operative correction of hypokalemia and successful anaesthetic and fluid and electrolyte management of the patient are discussed.

## INTRODUCTION

Bartter’s Syndrome is a rare disease associated with defective ion transport in renal tubules. Hypokalemic metabolic alkalosis is the hallmark of the condition along with effects on renin angiotensin aldosterone axis. Clinically, the patient may have reduced growth and dehydration. We have discussed the management of a child with Bartter’s syndrome with gall stones presenting for laparoscopic cholecystectomy.

## CASE REPORT

An 8-year-old, 17-kg girl child suffering from Bartter’s syndrome (BS) with a history of gall stones was posted for laparoscopic cholecystectomy. She had been diagnosed with BS 1.5 months before and was on oral potassium chloride. A history of maternal polyhydramnios and premature birth was present. No haemolytic and metabolic disorders that could contribute to gall stone formation were found. History of excessive fatigue during physical stress, poor growth and increased thirst was present. Serum potassium during the first evaluation was 2.9 mmol/l with increased 24-h urinary potassium and chlorides [Table 1]. Electrocardiogram and renal and liver functions were within normal limits. The younger sibling of the patient was also suffering from BS.

**Table 1:** Serum electrolyte concentrations

Time of estimation	Serum electrolytes (all in mmol/l except Ca, in mg/dl)				
	Na	K	Cl	Ca	Mg
45 days before(during diagnosis)	130	**2.3**	90	9.2	2.5
Pre-operative day	146	3.5	101		2.7
Pre-operative	135	**2.9**	104		
At peak pneumoperitoneum	140	**2.9**	109	8.9	2.7
Post-operative 10 min	137	**2.3**	91	10	2.4
3 h	141	3.6	107	9.0	3.0
6 h (oral diet)	139	3.4	106	9.1	2.8
Day 2 morning (oral diet and K)	134	3.4	101		
Day 2 evening (Oral diet and K)	135	3.4	97		
Follow-up at 3 weeks	144	**1.8**	111	7.7	3.6

Reductions in K values are in bold

Pre-operatively, serum potassium was 3.5 mmol/l but urinary potassium and chloride remained high [Table 1]. The patient was premedicated with oral trichlofos and ondansetron. Her arterial pressure (BP) was 108/60 mmHg and pulse 88/min. We started two intravenous infusions: one of 10 mmol of potassium chloride in 250 ml of Ringer lactate, at 40 ml/h, and second of normal saline, for maintaining hourly volume requirement. Preoxygenation was followed by induction with inj. propofol 40 mg with 1% lignocaine; endotracheal intubation was performed after administering succinyl choline 30 mg; anaesthesia was maintained with oxygen-nitrous oxide (33%:66%), vecuronium, sevoflurane 0.5-1 % and IPPV to maintain end-tidal CO_2_ (EtCO_2_) of not less than 5.3 kPa. Entry points of laparoscopic cannulae were infiltrated with 0.25% bupivacaine. The patient was positioned for cholecystectomy (anti-Trendelenberg of 20° and left tilt of 15°). Monitoring consisted of pulse oximetry, non-invasive blood pressure, ECG, temperature, capnography, urine output, blood gas analysis and electrolytes. Nasogastric tube, Foley’s catheter and radial arterial line were inserted after induction.

Haemodynamics were stable intra-operatively but for increased heart rate and BP during the establishment of pneumoperitoneum which needed increased sevoflurane concentration and temporary hyperventilation. The ABG sample showed slight alkalosis and the rate of ventilation was adjusted to maintain EtCO_2_ of 5.3 kPa. Intra-abdominal pressure was limited to 1.87 kPa (14 mmHg). Neuromuscular blockade reversal and extubation were uneventful. Rectal diclofenac (25 mg) was inserted for post-operative analgesia. The procedure was completed in exactly 55 min and the patient was repositioned flat on the operating table. BP and heart rate were normal and the respiratory rate was 16 per min. Ten minutes after extubation, serum potassium was 2.3 mmol/l with mixed metabolic and respiratory alkalosis [Table 1 and 2]. The rate of infusion of the Ringer lactate and potassium chloride solution was increased (60ml/h, so as to provide 2.4 mmol/h of potassium). Haemodynamic parameters remained normal subsequently and the respiratory rate stabilized at 14 per minute. ABG after 3 and 6 h showed persistent metabolic alkalosis, with normal potassium levels (3.6 mmol/l and 3.4 mmol/l). The urine output was adequate throughout; the patient was allowed liquids orally after 6 h. Oral potassium chloride was resumed. The patient was discharged on the second day, without complications. Gall stone analysis showed the presence of organic matter, calcium, bile pigment, traces of carbonate and cholesterol. At 3-week follow-up, serum potassium was 1.8 mmol/l with no symptoms; oral potassium chloride was increased as per paediatrician’s advice.

**Table 2:** Arterial blood gas values

Time of estimation	ABGs				
	pH	pO_2_(kPa)	pCO_2_ (kPa)	HCO_3_ (mmol/l)	BE
Intra-operative (at peak pneumoperitoneum)(EtCO_2_: 5.58 kPa)	7.48	25.8	5.64	30.0	4.8
Post-operative (10 min)	7.57	12.9	4.45	32.3	8.4
3 h post-operative	7.51	12.8	5.32	33.4	9
6 h post-operative (oralfluids, oral K)	7.52	13.1	5.98	36.8	12.8
Follow-up at 3 weeks	7.58	11.8	4.57	33.7	10

## DISCUSSION

BS is an autosomal recessive disease, with an incidence of 1.2 per million.[1] Classic BS described by Bartter in 1962 is the type III disease associated with defect in ion transport in the thick segment of the ascending loop of Henle and distal convoluted tubule.[2,3] This results in increased urinary loss of potassium and hypokalemic metabolic alkalosis with hypercalciuria.[4] Loss of sodium and chloride stimulates the renin-angiotensin-aldosterone axis. Aldosterone stimulates the secretion of potassium with sodium uptake, precipitating hypokalemia and secretion of hydrogen ions, and metabolic alkalosis. The increased secretion of atrial natriuretic peptide may also be a ‘cause’ of the syndrome.[5] Renal function may deteriorate over years. The metabolic alkalosis can shift the oxyhaemoglobin dissociation curve to the left, increasing affinity of oxygen to haemoglobin and reducing oxygen delivery to the tissues. It can also contribute to decreased ionic calcium and seizures.[4]

Children with BS have poor growth, dehydration, polyuria, polydipsia or diarrhoea but normal mental development.[2–4,6] The child reported here had poor growth for age (weight:17 kg; less than the fifth percentile for a height of 122 cm), with history of increased thirst, frequent diarrhoea along with history of polyhydramnios and prematurity, typically seen in BS.[3,7,8] The disease was confirmed by the presence of hypokalemia and increased urinary potassium. The child was advised high water intake at home along with fruit juices to avoid dehydration and to maintain urine output. Hypokalemia can be corrected by potassium-rich diet and by oral potassium chloride supplements (potassium chloride10%) which is safe for long-term use, at doses from 1 to 5 mmol/kg/day, up to 200 mmol/day.[6] Based on this, 10-15 ml of the liquid (12-18 mmol of potassium) four times a day was advised to be taken by the patient. For acute improvement, iv. infusions can be given, at 0.5 mmol/kg/h, at concentrations of up to 40 mmol/l by the peripheral vein (up to 60 mmol/l via central veins, with severe deficits).[3,6] We started the patient on intravenous potassium to have a better peri-operative control.[3,6,9,10] Dextrose and dextrose normal saline were avoided peri-operatively as they can precipitate hypokalemia. With Ringer lactate being otherwise an ideal solution for maintenance (even though the potassium concentration is less than 5 mEq/l), potassium chloride was added to this solution as per the recommendations discussed above. Additional fluid requirements were made up with normal saline as per the Holliday and Segar’s formula.

Normotension (as in the present case) or low BP with attenuated response to vasopressors is possible in BS due to increased renal prostaglandin E2 levels, in spite of the activation of the renin-angiotensin-aldosterone axis.[2,6] Hypokalemia, hypovolemia and positive pressure ventilation can add to haemodynamic instability.[9] Metabolic alkalosis may get corrected with the correction of hypokalemia. Increased renal prostaglandin E2 may respond to the administration of non-steroidal anti-inflammatory agents/COX2 inhibitors.[2,3,7] Other drugs used include potassium sparing diuretics, aldosterone antagonists and ACE blockers.[5]

Estimations of serum renin, aldosterone and prostaglandin E could not be done as facilities were not available. High urinary potassium levels indicate renal potassium losses.[6] Gitelman’s syndrome (with hypomagnesaemia), chronic diuretic or laxative abuse,[3,11,12] chronic vomiting, congenital chloride diarrhoea are differential diagnoses for BS.[13]

There are very few reports of children with BS undergoing anaesthetic exposures. Successful anaesthetic management of an 8-year-old boy with BS and bronchial asthma for bilateral orchidopexy under caudal epidural analgesia has been described.[1] Gall stones in a child affected with BS is even more uncommon. A Medline search opened up only one result of a 6-month-old boy found to have gall stones on ultrasound examination, having spontaneous drainage of the stone.[8] Chronic dehydration and metabolic alkalosis in BS could contribute to gall stone formation.[8]

Pre-operative sedatives/analgesics are needed to reduce anxiety and pain induced precipitation of alkalosis. The optimisation of electrolytes, including intravenous infusion of potassium, may be necessary.[1,14–16] Previously published papers report increases in serum potassium by increase in oral potassium and by iv. supplements.[1,14] Chronic hypokalemia is unlikely to be associated with dyrrhythmias; safe management of anaesthesia for 6 h in a patient with BS with 2.9 mmol/l potassium, without potassium supplementation, has been reported.[17]

A child with undescended testis for orchidopexy was successfully managed solely under caudal epidural analgesia, using bupivacaine 0.5%.[1] We followed the standard anaesthetic protocol in the present case using propofol, succinyl choline, oxygen-nitrous oxide, vecuronium and sevoflurane and IPPV, along with infiltration of port sites with bupivacaine. We avoided halothane because of its dyrrhythmic potential in the presence of hypokalemia and increased arterial CO_2_ tension (PaCO_2_) during pneumoperitoneum. Anti-Trendelenberg position, IPPV and raised intra-abdominal pressure can contribute to fall in BP with a reduced venous return. Hence, constant monitoring of haemodyamics along with capnography, arterial blood gas estimation and electrolytes is important. BP and heart rate variations in the awake state and after general anaesthesia in a patient of BS posted for renal biopsy have been investigated.[9] The authors caution that the combination of prostaglandins, hypovolemia, hypokalemia, halothane, nitrous oxide and IPPV can lead to unstable baroreceptor function. Pre-operatively, we had satisfactorily corrected hypokalemia, avoided hypovolemia with oral and intravenous fluids, avoided halothane and maintained ventilation based on end-tidal CO_2_ and ABGs, thus pre-empting this risk. Similar precautions were ensured in intra- and post-operative periods. In severe forms of BS, there can be skeletal muscle weakness and exaggerated response to relaxants; our patient behaved normally.

Serum potassium 10 min post-extubation was 2.3 mmol/l, with metabolic alkalosis; hence the rate of potassium supplementation was increased. After 3 and 6 h, potassium levels were corrected, with persistent metabolic alkalosis. The infusion of potassium was replaced by oral potassium. The next day, electrolytes had normalised and the child was discharged. At 3-week follow-up, the child still had significant hypokalemia and metabolic alkalosis, indicating persisting pathology of the disease. An increase in oral potassium supplements was advised.

In conclusion, understanding the pathological basis of BS can minimize morbidity, irrespective of the type of anaesthesia. Surgeries in children with BS are rare; we believe ours is the first reported case of a child with BS for laparoscopic cholecystectomy, which could be managed successfully by proper preoperative optimisation of electrolytes and fluid status and close peri-operative monitoring.
